# Sprouty 2 Is an Independent Prognostic Factor in Breast Cancer and May Be Useful in Stratifying Patients for Trastuzumab Therapy

**DOI:** 10.1371/journal.pone.0023772

**Published:** 2011-08-31

**Authors:** Dana Faratian, Andrew H. Sims, Peter Mullen, Charlene Kay, InHwa Um, Simon P. Langdon, David J. Harrison

**Affiliations:** Edinburgh Breakthrough Research Unit and Division of Pathology, Institute of Genetics and Molecular Medicine, University of Edinburgh, Edinburgh, Scotland, United Kingdom; Yale Medical School, United States of America

## Abstract

**Background:**

Resistance to trastuzumab is a clinical problem, partly due to overriding activation of MAPK/PI3K signalling. Sprouty-family proteins are negative regulators of MAPK/PI3K signalling, but their role in HER2-therapy resistance is unknown.

**Patients and Methods:**

Associations between Sprouty gene expression and clinicopathological features were investigated in a breast cancer microarray meta-analysis. Changes in expression of Spry2 and feedback inhibition on trastuzumab resistance were studied in SKBr3 and BT474 breast carcinoma cell lines using cell viability assays. Spry2 protein expression was measured by quantitative immunofluorescence in a cohort of 122 patients treated with trastuzumab.

**Results:**

Low gene expression of *Spry2* was associated with increased pathological grade, high HER2 expression, and was a significant independent prognostic factor. Overexpression of Spry2 in SKBr3s resulted in enhanced inhibition of cell viability after trastuzumab treatment, and the PI3K-inhibitor LY294002 had a similar effect. Low Spry2 expression was associated with increased risk of death (HR = 2.28, 95% CI 1.22–4.26; p = 0.008) in trastuzumab-treated patients, including in multivariate analysis. Stratification of trastuzumab-treated patients using PTEN and Spry2 was superior to either marker in isolation.

**Conclusion:**

In breast cancers with deficient feedback inhibition, combinatorial therapy with negative regulators of growth factor signalling may be an effective therapeutic strategy.

## Introduction

Although the HER2-targeting receptor tyrosine kinase (RTK) inhibitor trastuzumab (Herceptin) has clinical efficacy in both early and metastatic breast cancer, measurement of HER2 protein expression or gene amplification status is a relatively poor predictor of response with a low positive predictive value [1], [2]. The documented benefit of adjuvant trastuzumab combined with chemotherapy versus chemotherapy alone in terms of overall survival in HER2 positive patients is modest (96% vs 95% respectively at 1 year [1] and 91% vs 87% respectively at 4 years [2]). A large proportion of patients therefore unnecessarily receive ineffective and expensive treatments with possible toxic side-effects. Mechanisms of resistance must be elucidated in order to more efficiently select patients who will respond to therapy. Suggested mechanisms of *de novo* and acquired resistance to trastuzumab include *PIK3CA* activating mutations, *PTEN* inactivation, *IGF1R* over-expression and expression of p95 HER2 isoforms [3]–[5]. Although much attention has been paid to ‘forward-signalling’ mechanisms of pathway activation such as activating mutations in cellular oncogenes (eg *RAS*, *RAF* or *PIK3CA*), it is as likely that loss of negative feedback control also causes aberrant pathway activation, as is the case with mutation or decreased protein expression of PTEN. We hypothesised that one of the best characterised and potent EGF-induced negative feedback regulators, the Sprouty-family of proteins [6]–[12], may also be activated as a feedback inhibition programme downstream of HER2 receptor, and therefore contribute to sensitivity or resistance to trastuzumab.

To date there have been no reports implicating Sprouty in therapeutic sensitivity or resistance. The only published report of expression of Sprouty in breast cancer showed decreased expression at transcript level of Spry1 and Spry2 in 78% and 96% respectively of a small panel of breast cancers (n = 50) [13]. In spite of persistent attempts to establish the underlying mechanism for decreased expression, the exact cause remains elusive and may be different for specific orthologues in different cancers. In prostate cancer, there is conflicting evidence regarding the epigenetic regulation of *Spry1*, *Spry2* and *Spry4*, with some authors showing that *Spry2* and *Spry4* are downregulated by hypermethylation [14], [15], although in a separate study no hypermethylation of the promoter region of *Spry2* was identified [16]. Likewise, loss of heterozygosity (LOH) of *Spry2* on chromosome 13 has been found in prostate cancer [14], but not in other cancers. In breast cancer, none of the Sprouty family members are downregulated by either LOH or epigenetic mechanisms [13]. Given the dynamic nature of Sprouty expression in response to ligand drive, it is possible that detection of low expression levels reflects the activation state of the signalling network rather than a genetic or epigenetic phenomenon.

Our objectives were to (1) investigate whether Sprouty 2 expression is associated with established clinicopathological parameters, including prognosis, in breast cancer, and (2) establish what role, if any, Sprouty 2 expression levels play in therapeutic resistance and sensitivity to trastuzumab.

## Methods

### Ethics statement

The study was approved by the Lothian Research Ethics Committee (08/S1101/41). No informed consent (written or verbal) was obtained for use of retrospective tissue samples from the patients within this study, most of whom were deceased, since this was not deemed necessary by the Ethics Committee, who waived the need for consent. All samples were anonymised.

### Gene expression microarray meta-analysis of Sprouty 1, 2 and 4

A meta-analysis of six Affymetrix gene expression datasets comprising a total of 1,107 primary human breast cancers was performed as previously described [17]. Patient grade and follow-up information was retrieved from the original studies [18]–[23], and clinicopathological characteristics for the dataset are summarised in Table 1. The follow-up endpoints for the Chin *et al.*, Pawitan *et al.* and Sotoriou *et al.* datasets were recurrence-free survival and for Desmedt *et al.*, Ivshina *et al.* and Wang *et al.* datasets it was disease-free survival. Gene expression levels of Sprouty family genes were also investigated in the datasets of Chen *et al.* and Lu *et al.* to compare gene expression with normal breast tissue and HER2 immunohistochemical status, respectively [24], [25]. The Affymetrix probesets studied were SPRY1 (212558_at), SPRY2 (204011_at), SPRY4 (221489_s_at), HER2 (216836_s_at).

**Table 1:** Clinicopathological characteristics of patients analysed in this study.

Cohort variable		Cohort 1			Cohort 2	
	*Number*	*Percentage*	*Log-rank p-value*	*Number*	*Percentage*	*Log-rank p-value*
**Age, years**			0.30			0.46
<50	263	23.8		49	40.1	
>50	398	36.0		73	59.9	
NK	446	40.3		0	0	
**NPI**						0.22
<3.4	-	-		2	1.6	
3.4–5.4	-	-		47	38.5	
>5.4	-	-		62	50.8	
NK	-	-		11	9.0	
**Grade**			**<0.0001**			0.80
1	167	15.1		1	0.8	
2	330	29.8		19	15.6	
3	287	25.9		99	81.1	
NK	323	29.2		3	1.6	
**Tumour Stage**			**<0.0001**			**0.024**
1	338	30.5		35	28.7	
2	309	27.9		64	52.5	
3	15	1.4		12	9.8	
4	445	40.2		3	2.5	
NK	0	0		8	6.6	
**Node stage at diagnosis**			**<0.0001**			0.20
Negative	780	70.5		26	21.3	
Positive	157	14.2		87	71.3	
NK	170	15.4		9	7.4	
**Molecular phenotype**			0.061			
Basal	172	15.5		-	-	
Luminal A	336	30.4		-	-	
Luminal B	161	14.5		-	-	
HER2	194	17.5		-	-	
Normal-like	244	22.0		-	-	
**ER status**			0.220			**0.038**
>3	239	21.6		72	59.0	
≤3	700	63.2		41	33.6	
NK	168	15.2		9	7.3	
**HER2 status**						0.38
Positive	-	-		90	73.7	
Negative	-	-		32	26.3	
NK	-	-		0	0	
**Chemotherapy**						**<0.0001**
Anthracycline-containing	-	-		66	54.1	
Taxane-containing	-	-		53	43.4	
NK	-	-		3	2.5	

Cohort 1 is the gene expression cohort, and cohort 2 is the trastuzumab-treated cohort. NPI = Nottingham Prognostic Index.

### Cell culture

Cell lines were obtained from ATCC. SKBr3 and BT474 breast adenocarcinoma cell lines were grown as monolayer cultures in DMEM supplemented with 10% heat-inactivated foetal calf serum (FCS) and penicillin/streptomycin (100 IU/mL) in a humidified atmosphere of 5% CO_2_ at 37°C.

### Constructs, transfection, and cell viability

The FLAG-hSpry2 and FLAG-HSpry2^Y55F^ constructs were a kind gift from Dr Graeme Guy (Signal Transduction Laboratory, Institute of Molecular and Cell Biology, National University of Singapore) and used as previously described [26], [27]. In addition, empty pXJ40FLAG vector was constructed by digesting hSpry2-containing pXJ40FLAG vector at BamH1 and BglII restriction sites. Both mutant and normal sequences were verified by DNA sequencing, and empty vector confirmed by gel electrophoresis. At 70% confluence, cells were transfected with 1–2 µg of FLAG-tagged plasmid DNA using Lipofectamine 2000 reagent (Invitrogen) according to the manufacturer's instructions. On the following day, the cells were trypsinised and plated into 96-well plates at a concentration of 1000 cells/well. The cells were treated with or without trastuzumab (10 µg/ml) for 24 or 48 h. Cell viability was measured using the AlamarBlue reagent (AbD Serotec), according to manufacturer's instructions.

### Samples and tissue microarray construction

The population characteristics of the retrospective trastuzumab-treated cohort are summarised in Table 1 and have been described previously [28]. *HER2* gene amplification status was determined by fluorescence *in situ* hybridisation (FISH; DAKO HER2 FISH PharmDx, Ely, Cambridgeshire). Overall survival was calculated from date of initial diagnosis to date of death by any cause. Following H&E sectioning of representative tumour blocks, tumour areas were marked for TMA construction and 0.6 mm^2^ cores placed into 3 separate TMA replicates for each sample, as previously described [29].

### Immunofluorescence and AQUA automated image analysis

A detailed description of the AQUA HistoRx methodology is available elsewhere [30], [31]. Briefly, slides were incubated with primary antibodies diluted in 0.025% PBST for 1 h at room temperature (AE1/AE3 mouse monoclonal cytokeratin antibody, rabbit polyclonal to hSpry2 (Novus Biologicals diluted 1∶100 and 1∶25 respectively). Pan-cytokeratin antibody was used to identify infiltrating tumour cells and normal epithelial cells, DAPI-counterstain to identify nuclei, and Cy-5-tyramide detection for target (hSpry2) for compartmentalised (tissue and subcellular) analysis of tissue sections. Antibody specificity for hSpry2 antibody was determined by a single band on western blot, positive tissue controls, and localisation in the epithelial compartment, together with omission of primary antibody as a negative control. Only invasive tumour areas were included in the analysis; areas of *in situ* disease or normal epithelium were excluded by masking prior to analysis.

### Study design and Statistics

REMARK guidelines were adhered to where possible [32]. The biomarker analysis was a retrospective cohort study, with a fixed sample sizes and the study not designed to detect an overall effect size. No stratification or matching were used. Both cohorts used within this study have been described elsewhere [17], [20]. Median follow up for the gene expression metadata was 7.4 years (range 0–23.9 years) and the trastuzumab-treated cohort 1.8 years (range 0–66.8). Comparison of gene expression groups were by Mann-Whitney test for two independent groups and Kruskal Wallis test for more than two groups. AQUA scores were averaged from replicate cores, and cores containing <5% malignant epithelium were excluded. We used the software programme, X-Tile, to determine the optimal cutpoint while correcting for the use of minimum *P* statistics [33], which is known to inflate type I error when used incorrectly [34]. Two methods of statistical correction for the use of minimal *P* approach were utilised: the first by calculation of a Monte Carlo *P*-value and the second using the Miller-Siegmund minimal *P* correction [34]. Overall survival was subsequently assessed by Kaplan-Meier analysis with log-rank for determining statistical significance. Relative risk was assessed by the univariate and multivariate Cox proportional hazards model. All calculations and analyses were two-tailed where appropriate and performed using SPSS 14.0 for Windows (SPSS, Inc., Chicago IL).

## Results

### Spry2 is differentially expressed across clinicopathological subgroups of breast cancer and is an independent prognostic factor

**Figure 1:**
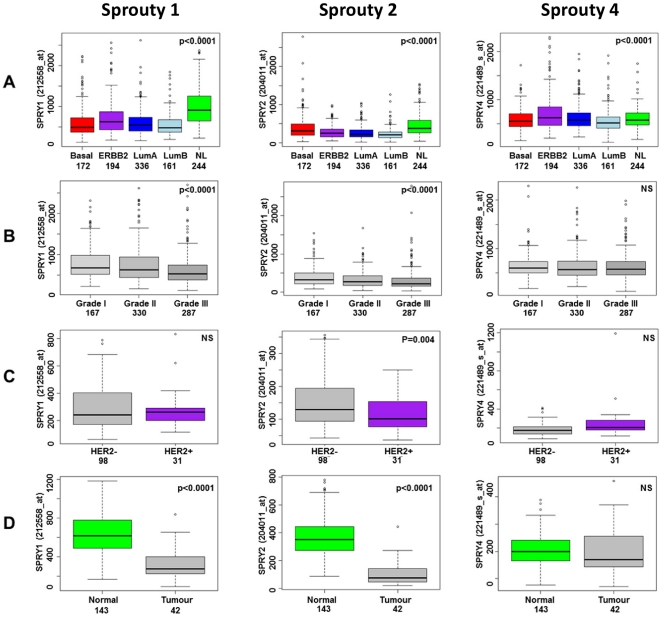
Gene expression of Sprouty-family members in relation to clinicopathological parameters; subtype (A), grade (B), HER2 status (C) and compared to normal breast tissue (D) in a meta-analysis of 1107 breast carcinomas [**17**] (A and B) or in single datasets (C and D) [**24]**, [25****].

**Figure 2:**
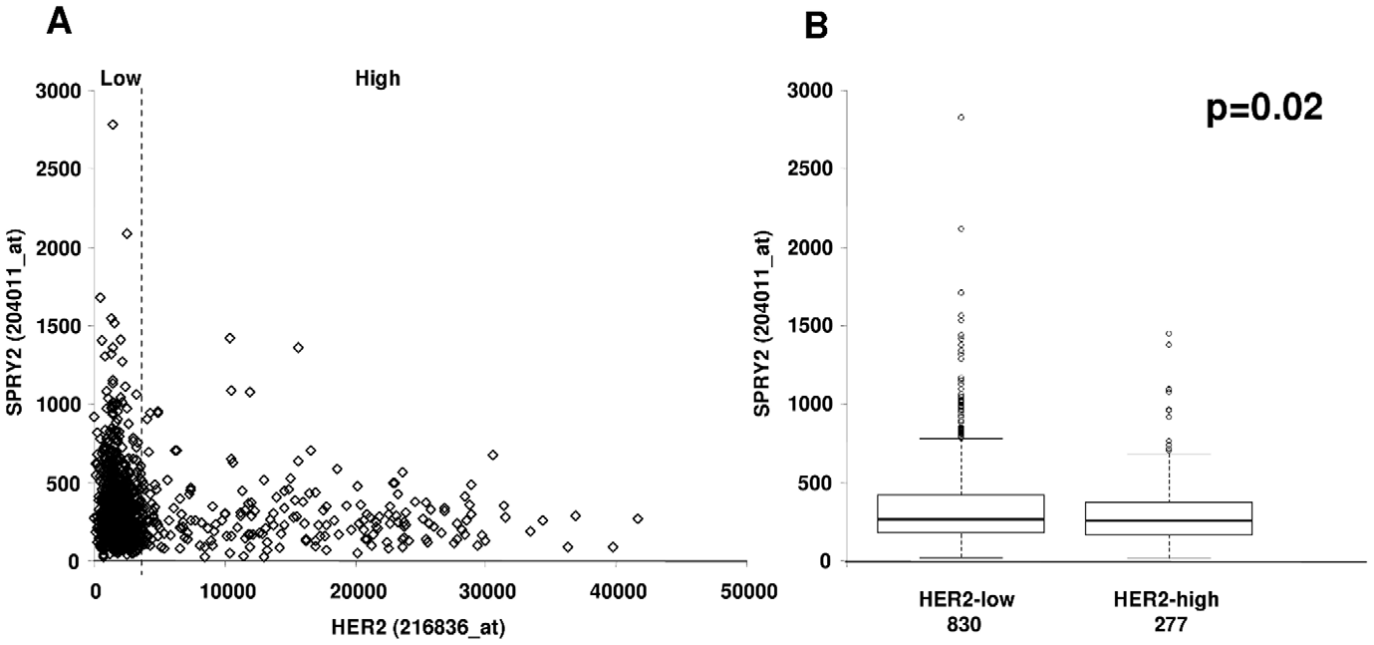
Relationship between *HER2* and *Spry2* gene expression. *Spry2* shows a wide range of expression in both HER2 high and low (split at the upper quartile) expressing tumours (A). On average, tumours expressing high levels of HER2 have decreased *Spry2* expression levels (B).

We first performed a meta-analysis of six published breast cancer gene expression profiles representing a total of 1107 tumours to assess the gene expression of *Spry1*, *Spry2* and *Spry4*. *Spry3* was omitted from the analysis since this is considered a minor orthologue, transcript levels are low across published datasets, and it is not represented on the Affymetrix U133A GeneChip. Sprouty family genes were differentially expressed across the five intrinsic breast cancer subtypes [35], with high expression of *Spry 1* and *Spry2* in normal-like cancers and higher expression of *Spry4* in basal-like and normal-like cancers (Figure 1A). Higher grade tumours had lower expression of *Spry1* and *Spry2* (Figure 1B). To investigate the association of Sprouty transcript with clinicopathological variables further, we analysed two further gene expression datasets. *Spry2* gene expression was found to be lower in a panel of invasive ductal carcinomas compared to normal breast tissue, and lower in HER2-positive (by immunohistochemistry) tumours (Figures 1C and 1D). Although the tumours in the meta-dataset did not have individual HER2 IHC status, separating them according to an upper quartile gene expression cut-point (25% ‘HER2-high’) confirmed that tumours with high expression of HER2 have significantly (p = 0.02) lower Sprouty 2 (Figure 2). Although the highest *Spry2* expression levels were observed in those tumours with low *HER2* gene expression (Figure 2), there was still a wide range of expression of *Spry2* in HER2-high tumours (Figure 2). We therefore speculated that Sprouty 2 could have an impact on therapeutic response to trastuzumab and act as a potential predictive factor.

**Figure 3:**
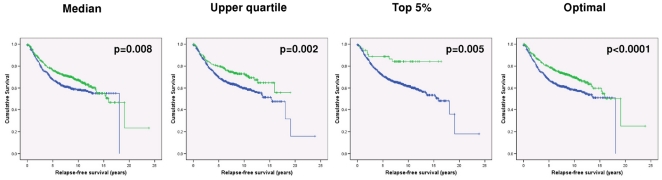
Prognostic significance of *Spry2* expression. Patients with high expression (green lines) of *Spry2* have the best prognosis, with very high expressers showing the most favourable outcomes.

First, however, we were keen to determine whether Sprouty 2 could be used as a prognostic factor, independent of other clinicopathological parameters. High *Spry2* gene expression was consistently associated with better prognosis (optimal cutpoint HR 1.49, 96% CIs 1.21–1.84, p<0.0001), particularly in those tumours expressing very high levels of *Spry2* (HR 2.71, 95% CIs 1.34–5.46, p = 0.005), consistent with the accepted role of Sprouty 2 as a tumour suppressor gene (Figure 3). Higher stage, grade and node status were associated with poorer survival in univariate analysis (Table 1); in multivariate analysis, stage, grade and *Spry2* expression remained significant prognostic variables (*Spry2* HR 1.33, 95% CIs 1.02–1.74, p = 0.04; stage HR 1.4, 95% CIs 1.12–1.82, p = 0.005; grade HR 1.20, 95% CIs 1.00–1.44, p = 0.05). Sprouty 2 may therefore identity patients with a more favourable outcome, even when tumours exhibit poor pathological features.

### Spry2 expression acts synergistically with trastuzumab to reduce cell viability in vitro: Forced feedback inhibition with chemical inhibitors has a similar effect

**Figure 4:**
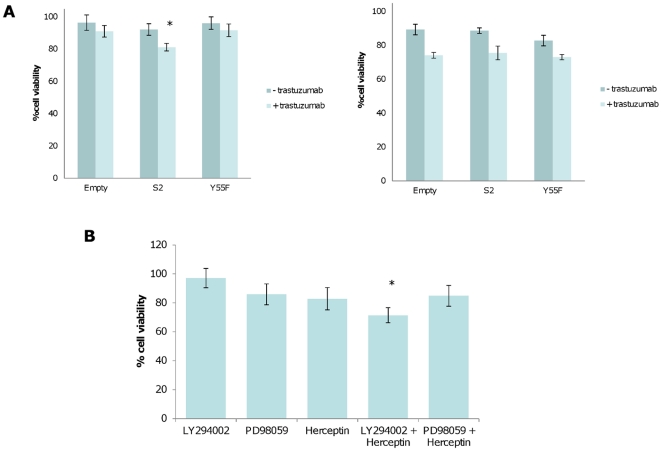
The effects of Sprouty 2 expression on response to trastuzumab *in vitro*. (A) Cell viability (AlamarBlue) assays to assess the effect of Spry2 on sensitivity to trastuzumab in trastuzumab resistant SKBr3s (left panel) and trastuzumab sensitive BT474s (right panel). Values are % cell viability compared to untreated controls. Expression of full length Spry2 results in a significant decrease in cell viability (asterisk, Student's t-test, p = 0.0008) compared to control or dominant negative Spry2^Y55F^. (B) Trastuzumab and LY294002 show synergistic inhibition of cell viability (asterisk, Student's t-test, p = 0.042) in trastuzumab-resistant SKBr3 breast cell lines.

Since *Spry2* was most closely associated with HER2 status, we next investigated what the effect of altering steady-state expression of Spry2 was on cell growth and therapeutic response, using transient expression of wild-type and dominant negative Spry2^Y55F^. Transfection efficiencies and endogenous expression levels are demonstrated in Figure S1. SKBr3 breast adenocarcinoma cell lines were insensitive to treatment with trastuzumab, while BT474s were sensitive at 48 h (Figure 4) when grown in full serum conditions. Overexpression of empty vector, Spry2, or Spry2^Y55F^ dominant negative construct resulted in no significant changes in growth in either of the cell lines at 48 h. However, overexpression of Spry2 significantly increased sensitivity to trastuzumab at 48 h in trastuzumab-insensitive SKBr3s, but there was no difference in growth in BT474s with either the full length or dominant-negative constructs. Re-establishing feedback inhibition in Sprouty-low tumours may therefore be an effective strategy for combinatorial therapy with trastuzumab, and raises the possibility that in some HER2 overexpressing tumours, high expression of Spry2 may be a marker of response to trastuzumab. We tested the combinatorial approach *in vitro* by substituting the negative feedback control of ERK and PI3K signalling of Spry2 with the chemical inhibitors LY294002 and PD98059, which inhibit PI3K and MEK1 respectively, with and without treatment with trastuzumab. As expected, trastuzumab showed little effect on cell viability alone, but a synergistic effect when SKBr3 cells were pretreated with LY294002, inhibiting growth by 29% at 24 hours (Figure 4). Forcing feedback inhibition through combinatorial approaches may therefore be a novel therapeutic strategy in tumours with *a priori* trastuzumab resistance.

### Low Spry2 expression is associated with poor outcome in trastuzumab-treated patients

**Figure 5:**
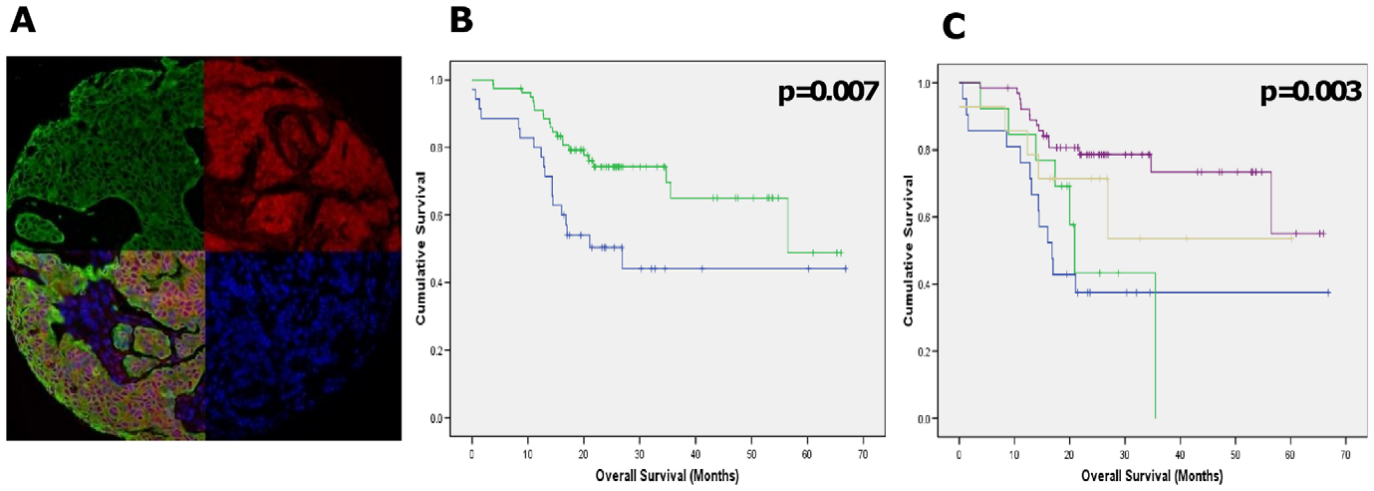
Quantitative expression of Spry2 is associated with trastuzumab sensitivity in patients. (A) AQUA fluorescent analysis of Spry2 expression in a tissue microarray core, showing cytoplasmic localisation of Spry2 (red) and masking of tumour areas for quantitation by cytokeratin (green). (B) Kaplan-Meier survival curves for patients treated with trastuzumab for low (blue) and high (green) protein expression of Spry2. (C) Kaplan-Meier survival curves for PTEN/SPRY2 high (purple), PTEN high (beige), Spry2 high (green) and PTEN/SPRY2 low (blue) patients. Overall survival is calculated from time of initial diagnosis to date of death.

Since higher levels of Spry2 were associated with increased therapeutic efficacy in the HER2+ SKBr3 breast cell line, we quantified expression of Spry2 in 122 primary breast tumours from patients who had been treated with trastuzumab using the AQUA fluorescence image analysis system ([28] and Figure 5A). This allowed us to test whether high expression levels of Spry2 protein were associated with clinical outcome in patients treated with trastuzumab in the clinical setting. The cut-point for Spry2 expression were calculated as described in the Materials and Methods, such that as well as showing high significance for difference in survival (p = 0.0069; Figure 5B), the cutpoint for Spry2 expression also maintained near significance with Monte Carlo simulations (p = 0.09) and correction for type I error (Miller-Seigmund p value = 0.12). In univariate analysis, tumour size, ER status, chemotherapy regimen, and Spry2 expression levels were all associated with significant survival differences (log-rank test, p<0.05, table 1), but Spry2 remained the only significant predictor of survival in multivariate analysis (Cox logistic regression, p = 0.002). Lymph node status was not significant in univariate analysis, most likely due to the low numbers of node-negative patients available for analysis in this high-risk population. High levels of Spry2 expression were associated with better overall survival than patients with tumours which expressed low levels of Spry2 (HR = 2.28, 95% CI 1.22–4.26; p = 0.008; mean survival 48 (95% CI 41–54 months) months vs 37 (95% CI 26–40 months) months for high and low Spry2 levels, respectively). This supports the role of Spry2 as a tumour suppressor gene in breast cancer, and its role in therapeutic resistance to trastuzumab.

Finally, since we have previously established that quantitative PTEN expression is also associated with outcome in the same cohort of trastuzumab-treated patients [28], and Sprouty 2 may exert some of its effects either directly or indirectly via PTEN [36], we reasoned that we could improve the predictive algorithm by considering the expression of both Sprouty 2 and PTEN. Protein expression of Sprouty 2 and PTEN were significantly correlated (Spearman's rank correlation coefficient 0.40, p<0.0001). In survival analysis, tumours expressing both high PTEN and high Sprouty 2 had the best outcome (mean survival 51 months), whereas those tumours expressing either PTEN or Sprouty 2 alone, or neither, had poorer outcomes (40, 24, and 32 months respectively). The relative risk of death in the Sprouty 2/PTEN high group was higher than either marker alone (RR 3.7; 95% CI 1.7–7.8, p = 0.001). When stratifying patients for trastuzumab therapy, there may therefore be increased value in combined measurement of pathway biomarkers.

## Discussion

The balance between positive and negative signals is critical in the maintenance of normal cell homeostasis in response to external stimuli, whether the stimulus is physiological (such as ligand drive) or therapeutic (such as with RTK or small molecule inhibitors of cellular signalling). The clinical implications of feedback control are becoming more readily appreciated. Loss of feedback inhibition in tumours treated with mTOR inhibitors (via increased expression of IRS-1) results in induction of AKT signalling, and may be responsible for the disappointing efficacy of mTOR antagonists in the clinic [37]. At worst, mechanisms such as unintended negative feedback contribute to the poor efficacy of agents when studied in Phase II and Phase III cancer trials and the high rate of attrition of drugs (approximately 30% due to efficacy), which is both time consuming and expensive [38].

Here we investigated the role of Sprouty-mediated feedback mechanism in breast cancer and its possible involvement in therapeutic resistance to RTK-inhibitors. In breast cancer, *Spry2* has been shown to be down-regulated at gene expression level compared to normal breast epithelium [13], which we confirmed in a meta-analysis of published gene expression data. Also consistent with its tumour suppressor function, *Spry2* expression decreases with increasing histological grade, and shows a strong association with relapse-free survival in a meta-analysis of over one thousand primary breast carcinomas, including in multivariate analysis. Sprouty 2 may therefore be a useful biomarker to stratify patients who are at very low risk of relapse and might not require adjuvant chemotherapy, even when there are other poor pathological prognostic features.

Since Sprouty expression was associated with HER2 status in our meta-analysis and has been shown to be expressed as a delayed early response (DER) gene downstream of other closely related growth factor receptors such as EGFR and FGFR, we further explored the association with HER2 in order to establish whether Sprouty plays an important role downstream of this therapeutically-targeted receptor. We explored the co-operativity of feedback by Sprouty on overcoming therapeutic resistance to trastuzumab by overexpressing Spry2 or dominant negative Spry2^Y55F^ in trastuzumab-resistant or sensitive cell lines expressing intermediate-levels of endogenous Spry2. Full length Spry2 synergised with trastuzumab to inhibit growth in trastuzumab insensitive SkBr3 cells. In some settings, therefore, reinstating negative feedback can overcome trastuzumab resistance. Since no Sprouty mimetics exist for therapeutic purposes, we used inhibitors of PI3K and ERK signalling, LY294002 and PD98059 in place of Spry2 feedback, since Spry2 can inhibit ERK directly or PI3K indirectly via PTEN [36]. LY294002, but not PD98059, synergised with trastuzumab to inhibit cell growth, suggesting that for cellular proliferation at least, inhibition through PI3K is the dominant synergistic feedback mechanism.

The link between Sprouty 2 expression and therapeutic response was further investigated in a clinical cohort of metastatic breast cancers treated with trastuzumab. Quantitative protein expression levels of Spry2 stratified patients for outcome in a series of 122 trastuzumab-treated breast cancers. Low Spry2 levels significantly correlated with decreased overall survival in multivariate analysis. Furthermore, when an integrated analysis of protein expression of PTEN and Sprouty 2 was performed, combined high expression of both biomarkers was superior to expression of each alone, or neither, in stratifying patients in the trastuzumab-treated cohort. This might reflect the role that Sprouty 2 plays in inhibiting PI3K signalling via PTEN [36]. Therefore, multiple biomarkers which capture multiple pathway control mechanisms may be superior to single biomarkers alone.

In conclusion, our data suggest that in a proportion of breast tumours deficient in negative feedback, combinatorial therapy with inhibitors of pathways downstream of RTKs may be an effective therapeutic strategy, and negative feedback proteins such as Sprouty may be useful biomarkers for selecting patients for these therapies.

## Supporting Information

Figure S1 **Transfection efficiency of S2 and Y55F constructs (A) and endogenous expression of Spry2 (B) in BT474 and SKBr3 breast cancer cell lines.** Cell lines were transiently transfected with increasing concentrations of DNA (measured in mg) in 6-well plates, and immunoblotted with anti-FLAG or anti-hSpry2 antibodies. Since endogenous expression was much lower than transfected expression, blots were re-probed with a longer exposure time (B) in order to compare protein expression of Spry2, which was similar in both cell lines.
(PPT)
