# Supplementary material for: Sprouty 2 Is an Independent Prognostic Factor in Breast Cancer and May Be Useful in Stratifying Patients for Trastuzumab Therapy
Source: PLoS One. 2011 Aug 31;6(8):e23772. doi: 10.1371/journal.pone.0023772 (PMC3166119; doi:10.1371/journal.pone.0023772)
Supplement: Figure S1 — Transfection efficiency of S2 and Y55F constructs (A) and endogenous expression of Spry2 (B) in BT474 and SKBr3 breast cancer cell lines. Cell lines were transiently transfected with increasing concentrations of DNA (measured in mg) in 6-well plates, and immunoblotted with anti-FLAG or anti-hSpry2 antibodies. Since endogenous expression was much lower than transfected expression, blots were re-probed with a longer exposure time (B) in order to compare protein expression of Spry2, which was similar in both cell lines. (PPT) [file pone.0023772.s001.ppt]

## Slide 1
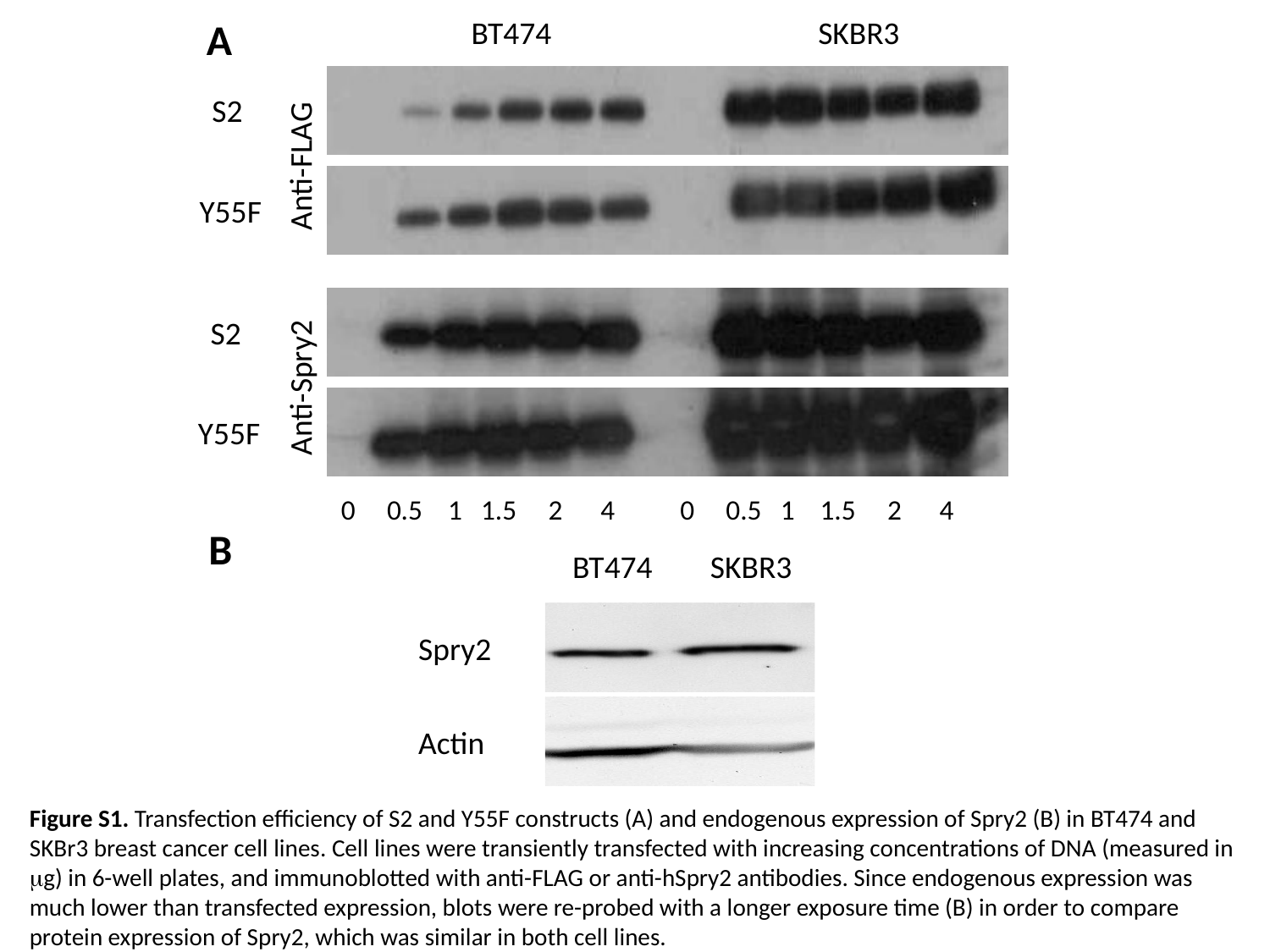

A
BT474
SKBR3
S2
Anti-FLAG
Y55F
S2
Anti-Spry2
Y55F
0 0.5 1 1.5 2 4
0 0.5 1 1.5 2 4
B
BT474
SKBR3
Spry2
Actin
Figure S1. Transfection efficiency of S2 and Y55F constructs (A) and endogenous expression of Spry2 (B) in BT474 and SKBr3 breast cancer cell lines. Cell lines were transiently transfected with increasing concentrations of DNA (measured in μg) in 6-well plates, and immunoblotted with anti-FLAG or anti-hSpry2 antibodies. Since endogenous expression was much lower than transfected expression, blots were re-probed with a longer exposure time (B) in order to compare protein expression of Spry2, which was similar in both cell lines.
